# Youth injury prevention in Canada: use of the Delphi method to develop recommendations

**DOI:** 10.1186/s12889-015-2600-x

**Published:** 2015-12-22

**Authors:** Ian Pike, Shannon Piedt, Colleen M. Davison, Kelly Russell, Alison K. Macpherson, William Pickett

**Affiliations:** University of British Columbia, F508-4480 Oak Street, Vancouver, BC V6H 3V4 Canada; British Columbia Injury Research and Prevention Unit, F508-4480 Oak Street, Vancouver, BC V6H 3V4 Canada; Queens University, Carruthers Hall, Office 203, 62 Fifth Field Company Lane, Kingston, ON K7L 3N6 Canada; University of Manitoba, 656-715 McDermot Ave., Winnipeg, MB R3E 3P4 Canada; York University, 337 Bethune College, 4700 Keele St, Toronto, ON M3J 1P3 Canada

**Keywords:** Modified-Delphi, Recommendations, Youth injury, Injury prevention

## Abstract

**Background:**

The Health Behaviour in School-aged Children Survey is one of very few cross-national health surveys that includes information on injury occurrence and prevention within adolescent populations. A collaboration to develop a Canadian youth injury report using these data resulted in, *Injury among Young Canadians: A national study of contextual determinants*. The objective of this study was to develop specific evidence-based, policy-oriented recommendations arising from the national report, using a modified-Delphi process with a panel of expert stakeholders.

**Method:**

Eight injury prevention experts and a 3-person youth advisory team associated with a Canadian injury prevention organization (Parachute Canada) reviewed, edited and commented on report recommendations through a three-stage iterative modified-Delphi process.

**Results:**

From an initial list of 27 draft recommendations, the modified-Delphi process resulted in a final list of 19 specific recommendations, worded to resonate with the group(s) responsible to lead or take the recommended action. Two recommendations were rated as “extremely important” or “very important” by 100 % of the expert panel, two were deleted, a further two recommendations were deleted but the content included as text in the report, and four were merged with other existing recommendations.

**Conclusions:**

The modified-Delphi process was an appropriate method to achieve agreement on 19 specific evidence-based, policy-oriented recommendations to complement the national youth injury report. In providing their input, it is noted that the injury stakeholders each acted as individual experts, unattached to any organizational position or policy. These recommendations will require multidisciplinary collaborations in order to support the proposed policy development, additional research, programming and clear decision-making for youth injury prevention.

**Electronic supplementary material:**

The online version of this article (doi:10.1186/s12889-015-2600-x) contains supplementary material, which is available to authorized users.

## Background

Injuries in children and youth range from minor inconveniences to major trauma, can limit the normal activities of daily living and have been recognized as an important health problem [1]. Common causes of injury to children and youth include falls, transport incidents, self-harm, struck by an object during sports, unintentional poisoning and violence [2]. As injury is the leading cause of death and hospitalization among young people in Canada, foundational epidemiological information is of great value to inform the development and targeted implementation of injury prevention and health promotion initiatives [1, 3].

One of the only cross-national health surveys that includes information on injury occurrence and prevention within adolescent populations is the *Health Behaviour in School-aged Children Survey* (HBSC) [4]. HBSC is an international survey conducted every four years in 43 countries that informs understanding of the behaviours and attitudes of youth ages 11–15 years, and the factors that impact their health [5]. Canada conducted its sixth survey cycle in 2010, with 26,078 students participating from 436 schools located in eight provinces and three territories. HBSC provides a rare and comprehensive glimpse into health problems, including injury, in the early adolescent years in Canada.

In the fall of 2011, members of the Canadian Institutes of Health Research (CIHR) Team in Child and Youth Injury Prevention, academic researchers associated with HBSC Canada, and the Public Health Agency of Canada met to collaborate on the development of a detailed plan for a Canadian youth injury report using HBSC data. Editorial teams were established, analyses were conducted and the report, *Injury among Young Canadians: A national study of contextual determinants,* was drafted [6]. The goal of the report was to use Canadian specific data from the 2009/2010 cycle of the HBSC to report nationally representative findings about injury occurrence, and the determinants and consequences of injury, particularly as these relate to the contexts where young people live, learn and play [6]. This was completed in order to support critical evidence-based initiatives to prevent injuries in this population. The development of specific recommendations was identified as a means to ascertain critical evidence-based actions based on the key insights illuminated by the report (Table 1). We approached this in a systematic and scientifically sound manner, using a modified-Delphi process. Delphi is a research tool for obtaining the judgment of a panel of independent experts on a specific topic [7], which facilitates a group communication process dealing with a complex problem [8]. The purpose of this component of the project was therefore, to develop specific policy oriented recommendations arising out of the report using a modified-Delphi process [7] administered to a panel of expert stakeholders.

**Table 1:** Key Findings from *Injury Among Young Canadians: A national study of contextual determinants*

Overall	• Factors related to the contexts, or environments, where young people learn, live and play have significant impact on their injury experiences.
	• Patterns for injury vary by important subgroups of youth. This suggests potential health inequities among youth, such as those who reside in group homes or in foster care, youth who are bullied, or youth living in rural settings.
	• Though it varies by age group and gender, at least one-third of all injuries are sports related, and one-half of all serious injuries are from driving or riding in a motor vehicle.
	• In both grades 6–8 and 9–10, boys report more injuries and more severe injuries than girls.
	• Individual behaviours and activities such as smoking, drinking, impaired driving and illicit or prescription drug use elevate the risk of injury.
Injuries at Home	• The proportion of youth reporting home injuries increased as their community size decreased.
	• Youth in foster care, in particular boys in grades 9–10, reported many more home injuries.
	• Going to school or bed hungry because there was not enough food at home was associated with home injury. Among boys in grades 9–10, those who frequently went to school or bed hungry were four times more likely to report being injured at home.
Socio-economic Status (SES)	• Individuals of lower SES report the highest incidents of injuries compared with those of average and high SES.
	• Youth attending schools in low SES neighbourhoods (those with high proportions of families with low income, less education or single parents) had a greater number of severe injuries.
	• Girls of lower SES in all grades had a far greater risk of school injury compared with girls of higher SES.
	• Boys in grades 9–10 living in neighbourhoods with high SES had a greater proportion of severe injuries than girls and younger boys.
School-based Injuries	• Taken together, physical activities such as training for a sport, bicycling, skating, walking and running are mechanisms for a third of all severe school injuries.
	• Boys and girls who were bullied reported higher proportions of school-based injuries, and had a greater risk of injury in all grades than those not bullied.
Neighborhood Characteristics	• Social characteristics, including a lack of trust within the neighborhood, fear of being taken advantage of by neighbours, feeling there are no good places to spend free time, and that the neighbourhood is not safe place to play, were associated with a greater likelihood of severe injury.
	• Physical characteristics of neighbourhoods that were associated with an increased likelihood of severe injuries included the absence of parks for boys and the presence of shabby buildings for girls.
Interactions with Peers	• Peers have a significant influence on a young person’s injury risk. Youth who did not engage in risk behaviours such as alcohol use or smoking were still at increased risk for injury if their peers engaged in these activities.
	• The proportion of youth who reported being injured increased as the frequency of participation in physical fighting increased.
	• The more frequently a young person communicated and spent time with friends, the greater their injury risk.
	• Among girls and younger boys, having close male friends increased the risk for injury.
Substance Use	• Injuries were more common among youth who reported either illicit drug use or the misuse of prescription drugs compared with those who did not use drugs.
	• Girls in grades 9–10 who used alcohol, prescription drugs or illicit drugs were more likely to be injured, and the greatest risk was associated with misuse of prescription drugs.
	• Among boys and girls, the percentage of injured youth increased as the frequency of binge drinking increased.
	• Youth who engaged in binge drinking reported sustaining more on- and off-road motor vehicle injuries than those who did not binge drink.
	• The proportion of those severely injured in an on- or off-road motor vehicle collision was approximately double for those who reported being an impaired driver or a passenger of an impaired driver compared with those who were not.
Rurality	• Youth who resided in rural areas reported more injuries per capita than their urban counterparts.
	• Boys in rural areas had the highest reports of driving a motor vehicle while drinking alcohol or using drugs.
	• Fifteen percent of girls from small urban centres also reported impaired driving which was considerably higher than girls from large urban centres.

## Methods

The national report had seven core chapters examining risk factors for injury, particularly within home, school, neighbourhood and peer-group contexts. Prior to the first round of the modified-Delphi process, the 22 different chapter authors of the report were asked to suggest 3–7 draft recommendations for each of their chapters. The draft recommendations intentionally reflected both positive and negative findings, the lesson(s) that could be learned and suggested actions to be taken. Upon receipt of 37 draft recommendations from chapter authors, the research team removed duplicates and consolidated similar recommendations, reducing the number to 27. The 5 editors of the report are the main authors of this manuscript and constituted the research team. The draft recommendations were subsequently edited to ensure consistency of language and style so that they were written as policy-oriented action statements directed at the appropriate group to take the recommended action(s). This set of draft recommendations formed the basis for expert review within the modified-Delphi process. The University of British Columbia/Children’s & Women’s Hospital Research Ethics Board discussed this project and conveyed that ethics approval was not required as the project goal was simply to document the process of developing recommendations with those who were considered colleagues of the research team.

### Modified-Delphi method

The Delphi process is based on the assumption that group judgments are more valid than individual judgments. Delphi is an appropriate technique when: 1) the problem being addressed does not lend itself to precise analytical techniques and would benefit from collective expert opinion, 2) the required experts may not have a history of communication and/or collaboration, 3) logistics do not support frequent or face-to-face meetings, 4) disagreements among experts may require mediation and anonymity and 5) variety of expert opinion has to be preserved and provided as feedback in the iterative process, avoiding domination by any one opinion [8].

**Fig. 1:**
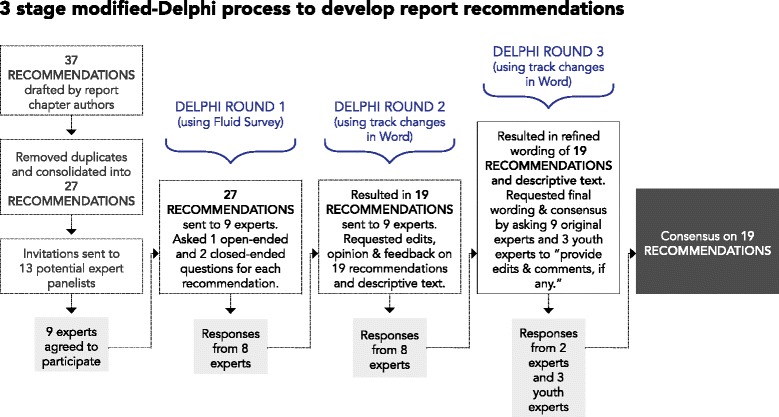
3 stage modified-Delphi process to develop report recommendations

For the purpose of developing specific recommendations in this study, a modified-Delphi method was chosen over traditional survey methods. This implied that: 1) experts were to be selected based on their unique ability to provide informed responses focused on the development of recommendations and 2) agreement was to be arrived at through the use of controlled and anonymous feedback provided by the facilitator during three rounds of review and feedback [7]. The Delphi process was modified here to be restricted to three iterative rounds of expert input, seeking ratings and comments using *FluidSurveys*™ [9] online survey software during the first round and subsequent input via email responses during the second and third rounds. The research team served in the role of facilitator, undertaking the synthesis between rounds. Mirroring the process used by Green et al. [10], the process of synthesis included discussion among the facilitator members, exploring all expert opinions, disagreements and suggestions for change, before synthesized recommendations were drafted for each subsequent round. The modified-Delphi process in this study was an iterative process aimed at agreement on a suite of evidence-based recommendations to support a preferred future of injury prevention for children and youth. The process was completed during the period May to September, 2013 (Fig. 1).

### Establishing the panel of expert stakeholders

An expert is defined as one who is considered to be knowledgeable about the subject under consideration and capable of representing the views of his or her peers [11]. As the report was national in scope, it was important to assemble a panel of injury prevention expert stakeholders who could represent all Canadian regions [12], though input from recognized international colleagues was also desired. In addition to geographic location, criteria for consideration included professional position and experience with youth injury prevention. The research team brainstormed a list of potential experts with positions in government, injury prevention organizations, public health, academic research, educational systems and youth-serving organizations as the youth injury report indicated required action and support by these groups. The injury prevention community in Canada is relatively small and stable, with many professionals having more than 15 years of experience in the field, therefore it was not difficult to brainstorm a list. Several Delphi studies recommend using 10–18 carefully selected expert respondents, enough to provide a range of opinions but also few enough for the research team to be able to summarize and integrate those opinions [13–16]. Thirteen experts were identified by the research team based on the stated criteria, including knowledge user stakeholders and researchers from the Alberta Centre for Child, Family and Community Research, Vancouver Island Health Authority, Saskatchewan Prevention Institute, IWK Health Centre (Nova Scotia), Simon Fraser University (British Columbia), Parachute Canada (National), Alberta Centre for Injury Control and Research, United States Centre for Disease Control, and Johns Hopkins Center for Injury Research and Policy (Maryland, USA). Experts each had between 15–36 years of experience in the injury prevention field and 10–25 years of experience in senior leadership positions that included a focus on knowledge translation. Of the 5 researchers identified, a combined list of their publications related to injury prevention program implementation, evaluation and policy analysis totaled 657. It was recognized, however, in providing their input that each injury expert was to act as an individual, with views that strictly speaking were unattached to any organizational position or policy. These individuals were not any of the 22 report chapter authors.

The research team recognized the need to ensure that youth contributed to the development of the recommendations. Therefore, the number of expert panelists was expanded in the third and final round to include 3 members of Parachute Canada’s Youth Advisory Team [17]. The Youth Advisory Team is a group of young Canadians aged 15–25, who are passionate about the issue of injury in Canada and who contribute to special projects and consultations.

### Conducting the modified-Delphi process

The nominated Principal Investigator of the CIHR Team in Child and Youth Injury Prevention extended personal invitations to the 13 potential expert panelists by email to participate in the development of the action-oriented specific injury prevention recommendations, and to inform them of the process and timeline [18, 19]. Nine individuals from injury prevention organizations, medical health centres, schools of public health and academic institutions agreed to participate.

#### Round 1

The goal of the first round of the modified-Delphi was to decrease the number of recommendations to a core group considered to be most important, and to improve the wording so that each recommendation would resonate with the group(s) responsible to lead or take the action. Experts were sent a survey using *FluidSurveys*™ [9] that provided the list of recommendations and a link to the report *Injury among Young Canadians: A national study of contextual determinants* [6]. Panelists were asked to respond to one open-ended [20] and two closed-ended questions for each of the recommendations: 1) Will this recommendation resonate with the group(s) responsible to lead or take the action (yes/no), 2) Do you see ways to improve the strength of the language, and if so, please re-write or add comments, and 3) Using a 3-point scale (extremely important, very important, or moderately important), how important is this recommendation.

When experts advised that a recommendation be deleted, merged with another or placed as content elsewhere in the report, the changes were made by the project facilitator. In a publication on the Delphi method, Okoli and Pawlowski, 2004, suggest retaining items selected by 50 % of the expert panelists [16]. This study was able to achieve a greater percentage of agreement and retained recommendations if 66 % of experts agreed that they would resonate with the group(s) responsible to lead or take the action. When comments conflicted, the research team acted as facilitator and used a process of discussion to reach a mutually satisfactory decision. Descriptive text that provided a rationale for each recommendation based on report findings was added in preparation for Round 2 of the modified-Delphi focused on the remaining 19 recommendations.

#### Round 2

The goal of the second round was to generate further expert opinion and feedback on the list of 19 recommendations resulting from Round 1. The same panel of experts was asked to use their expertise and background in child and youth injury prevention to consider the recommendations in light of the youth injury report. Using the ‘review’ and ‘track changes’ tool in the Microsoft Word software package, experts provided edits and comments to the recommendations, and submitted their edited version to the facilitator. All experts’ comments and edits were incorporated wherever possible. Where there were conflicting comments, they were reviewed by the facilitator and decisions were made on how to ensure that all comments were synthesized as part of the recommendations for inclusion in Round 3. All decisions and edits were documented and communicated back to the expert panel as part of the Round 3 recommendations that members could make final comment on.

#### Round 3

The goal of the third and final round was to finalize the wording of the recommendations. In addition to the same 9 expert panelists, members of the Parachute Youth Advisory Team were asked to submit their edits and comments, if any, using the ’review’ and ‘track changes’ tool within Microsoft Word, and to submit their edited version to the facilitator. The Youth Advisory Team were not included in all modified-Delphi rounds as the 3 round process was assessed by the facilitator as too time consuming for youth volunteers; however, 3 Youth Advisory Team members contributed to the final round. Their comments included suggested wording additions and excellent examples of injury incidents they were aware of that illustrated the importance of the recommendations. There was strong agreement regarding wording of the recommendations from expert panelists at the end of Round 3.

## Results

Responses to the 27 recommendations were received from 8 experts in the first round. Two recommendations were rated as resonating with the intended audience, and as “extremely important” or “very important” by 100 % of the expert panel: 1) *Federal and provincial Health Ministries as well as regional and community-level health organizations are urged to make investments to establish and continue surveillance efforts to identify new and emerging patterns of injury*, and 2) *School districts, schools and Parent Advisory Councils are encouraged to implement evidence-based peer-mentorship programs that address the social context of the school environment and improve feelings of belonging and safety.* Two of the recommendations received less than 66 % of the expert panel rating them as resonating with the intended audience, and less than 66 % of experts rating them as “extremely” or “very” important, and were subsequently deleted. The facilitator incorporated feedback on 2 other recommendations indicating that the content was important but should be placed as text elsewhere in the report, as it was not judged to be a recommendation.

Experts suggested that several recommendations could be merged due to their similar content, resulting in 4 being merged with existing recommendations. The remaining 19 recommendations were edited for consistency of language and style, based upon a total 135 open-ended question responses from the experts.

Two recommendations related to food security each received less than 66 % of expert ratings of resonance (57 % and 50 %, respectively) and importance (57 % and 63 %, respectively); however, a decision to retain the 2 recommendations was made based upon the expert panel opinions and the evidence revealed in the report regarding the relationship between injury and going to school or bed hungry [6]. While experts “applauded” the inclusion of these 2 recommendations, they were guarded in their assessments of the importance and how they would resonate with those who might lead and take action. Comments included: “To qualify - I hope that it will resonate”, “This is logical, so we don't need research- we need pilot interventions or communication so we address the hunger”, and “I really applaud you for including it”.

The second round again elicited responses from 8 expert stakeholders. Comments included requests for clarification related to confusing points, the addition of a rationale for 2 recommendations, and ensuring the recommendations were clearly based on evidence from the original youth injury report. For example, a reference to 4-sided pool fencing as a policy solution was deleted from the recommendations because swimming pools were not discussed in the report. Five expert panelists responded to the third and final round, three of whom were members of the Parachute Youth Advisory Team [17]. One of the expert panelists responded to indicate that she had no further comments. It is noted that panelists were asked to “provide edits and comments, if any”. Therefore, it is assumed that the remaining experts were content with the recommendations as written (Table 2).

**Table 2:** Final list of recommendations

Recommendation	Rationale
1. That federal, provincial, territorial and municipal governments, health and funding agencies make meaningful investments to support organizations that lead injury prevention initiatives in Canada.	Organizations that lead injury prevention efforts must be supported. In order to prevent injuries and enhance the lives of youth, it is necessary to change public attitudes around the acceptable frequency of injury events; the risk factors and causes of injury; the types and severities of injuries; the life-long impact that injuries can have; the time lost from education and other healthy activities; the pressure that injuries add to the health care system; the economic burden to society and families; and the fact that the vast majority of injuries are preventable.
2. That federal, provincial, territorial and municipal governments, health and funding agencies make meaningful investments to establish and continue comprehensive injury surveillance, inform injury prevention initiatives, monitor and evaluate outcomes and identify new and emerging patterns of youth injury.	Like chronic and infectious disease surveillance, it is critical that injury trends and patterns, which include social and contextual determinants, are monitored and evaluated over time. Efforts to collaborate and share injury data and information will increase understanding and the ability to take strategic actions to reduce and prevent injuries.
3. That federal, provincial, territorial and municipal governments review, establish and enforce regulatory and evidence-informed policy solutions to prevent and control injuries.	The role of government in injury prevention through the development and enforcement of good policy is essential. Examples include, but are not limited to, policies that promote training and safe operation of motor vehicles and off-road vehicles, reduction of impaired driving both on roads and in off-road situations, helmet use during wheeled activities, skiing, and snowboarding, and concussion prevention and management.
4. That federal, provincial, territorial and municipal governments, health and funding agencies implement policies and programs to minimize use of alcohol and drugs by youth. This includes efforts to address the culture that promotes alcohol and drug use, controlled regulation of alcohol sales, education on youth substance abuse and high-risk behaviours, and establishment of programs and services to address addictions.	Services, regulations and programs that address the use and abuse of alcohol, prescription and illicit drugs are critical. Substance use can lead to increased risk-taking and impairments that leave young people vulnerable to major injury. The recreational use of drugs and alcohol among passengers and drivers of off-road vehicles is a concern particularly in rural settings. Alcohol and drug use is also a marker for other lifestyle behaviours that lead to higher risk of injury.
5. That public health personnel, police liaison officers, Parent Advisory Councils, school districts, municipalities and other community and neighbourhood youth serving agencies continue to support and advocate for programs and policies aimed at preventing youth drug and alcohol abuse and promoting associated harm reduction programs as a protection against injury.	Injuries were more common among youth who reported illicit or prescription drug use, binge drinking or whose friends abused drugs or alcohol. Public health personnel, police liaison officers, Parent Advisory Councils, school districts, municipalities and youth servicing agencies can assist governments and funding agencies by advocating for, leading or supporting initiatives to address substance use.
6. That federal, provincial, territorial and municipal governments, health and funding agencies support policies, programs and services that increase food security and reduce family dysfunction.	This report has noted the relationship between injuries and youth of low socio-economic status or who go to school or to bed hungry. Among boys in grades 9–10, for example, those who reported often going to school or bed hungry were four times more likely to also report one or more home injuries. Efforts to reduce injury must be multifaceted and include research (see Recommendation 17 below), food security and other family support programs.
7. That injury researchers collaborate with medical and social welfare professionals to better understand injury risks and social disparity risk factors leading to home injuries, particularly among youth in foster care, and develop concrete recommendations for injury prevention initiatives based on this understanding.	Approximately 12 % of all youth injuries occur within the home and yard setting and not all youth are at equal risk of sustaining a home-based injury (see recommendation 6). A greater proportion of younger girls and older boys residing in group homes or foster care, for example, reported more injuries and severe injuries than their peers who were not in foster care. Injury prevention efforts need to be informed by greater understanding of the factors leading to these home injuries.
8. That Provincial and Territorial Education Ministries further integrate injury prevention education (e.g. bullying prevention, suicide prevention, drowning, burns, motor vehicle, sports injuries) into the school curriculum for grades 6 to 10 (and ideally from Kindergarten) and institute school policies to protect students from school-related injuries.	Because children and youth spend much of their time at school, examining the relationship between characteristics of the school setting and injury risk is extremely important. The school setting is an ideal place to integrate and evaluate injury prevention efforts, since children and youth can become involved in injury prevention initiatives at school, and many activities that can lead to injuries (e.g. playground use participation in school sports) occur in this setting.
9. That public health personnel, police liaison officers, Parent Advisory Councils, municipalities and other community and neighbourhood agencies review and understand the scope of youth injury in their community in order to implement and evaluate evidence-based injury prevention policies, programs and initiatives.	Every community has groups who are at higher risk for injury, such as youth from lower socio-economic status families or boys from affluent families who engage in high-risk sporting activities. The local injury stakeholders listed above need to work together with the researcher community to target and evaluate injury prevention initiatives towards high-risk groups.
10. That the Canadian Collaborating Centres for Injury Prevention and Parachute Canada partner and collaborate with Active Healthy Kids Canada, ParticipACTION, neighbourhood youth serving agencies and coaches to integrate injury prevention into the promotion of healthy physical activity for children and youth.	Physical activity and play are essential to healthy development among children and youth, but may be accompanied by increased risk of injury. Collaborations between those promoting physical activity and play, and injury prevention partners are essential to ensure safe, yet stimulating environments for healthy development. Examples include: redesigning school and community playing fields, play spaces and active transportation routes; informing school policies regarding bullying and supervision; providing return-to-play guidelines; providing sport-specific injury prevention; and integrating bullying interventions into physical activity promotion.
11. That municipalities, community and neighbourhood leaders, Parks and Recreation and schools work with youth to create safe physical environments where youth want to spend time.	Development of spaces for physical activity and play can positively influence sense of community and foster trust in neighbourhood, which is important for injury reduction. In addition, the provision of environments promoting physical activity can have positive influences on other health outcomes such as obesity.
12. That school districts, school administrators and Parent Advisory Councils review, continue to implement and support anti-bullying and anti-violence policies and programs that target perpetrators, victims and bystanders.	This report found that children and youth who reported being bullied were up to twice as likely to be injured than those who did not report being bullied. This association was particularly strong for boys in grades 6–8 and girls in grades 9–10. School administrators must lead in the delivery of appropriate consequences for bullying behaviours.
13. That school districts, school administrators, Parent Advisory Councils and youth leadership groups implement peer-mentorship programs that address the social context of the school environment and improve feelings of belonging and safety.	This report found a relationship between injury and emotional well-being, such as not feeling respected or not belonging at school. Schools must work together with local youth serving agencies, Parent Advisory Councils and youth to create a culture that fosters inclusiveness, respect and improves tolerance of differences/diversity and discourages bullying.
14. That research funding bodies support programs of research that seek to understand the fundamental determinants of youth injury, including surveillance to identify new patterns and trends, and interventions that target specific high risk and/or vulnerable populations, contextual determinants and risk and protective factors.	While this report provides some insights into the relationship between social and contextual determinants, risk and protective factors, and youth injury, more research is needed to enhance our understanding. Recommendations 15 to 19 are directed at the injury research community, including academic and applied researchers, policy makers and practitioners to develop innovative research projects that shed light on the following specific areas in relation to injury: substance use, high-risk youth, going to bed hungry, peer relationships and involvement in a sport club, youth club or voluntary service.
15. That research programs are developed and supported to improve understanding of the culture that promotes the use of alcohol, illicit and prescription drugs for recreational purposes, and the impact on child and youth injury patterns and rates.	As stated in recommendations 4 and 5, alcohol, illicit drug and prescription drug use are increasingly important risk factors for adolescent injury. Further research will aid our understanding of where and how to intervene.
16. That research programs are developed and supported to implement optimal methods to prevent injury among high-risk and/or vulnerable youth, including those from rural and remote regions; investigate recurrent determinants and patterns of injury; social disparities; and, risk and protective factors.	The risk of injury is not the same for all children and youth as some characteristics and behaviours increase or decrease risks for injury. In addition to level of family function, being bullied, involvement in high-risk sports, and use of alcohol, illicit and prescription drugs, this report found that the proportion of students reporting an injury increased as the population size of the city or town where they attended school decreased. Further research and evaluation is needed to inform targeted interventions for all high-risk children and youth, including those living in rural locations (<1,000 population).
17. That research programs are developed and supported to investigate the relationships between going to school or bed hungry and the increased risk for injury among youth, with a particular focus on policy solutions.	Understanding of the relationships between food insecurity, family dysfunction and injury among youth will allow solutions to focus on some of these root causes.
18. That research programs are developed and supported to illuminate understanding of the effects of various child and youth peer relationships and peer activities on injury risk.	The current findings suggest that there is merit in exploring social and contextual factors when creating injury prevention programming. Among girls, having close male friends increases the risk for injury; however, having close male friends is a protective against the occurrence of injury among older boys. As risk factors for injury vary for boys and girls, further research and evaluation is needed to inform targeted interventions.
19. That research programs are developed and supported to understand child and youth involvement in sports and social clubs as a protective factor against injury.	Children and youth involved in voluntary service, youth clubs, and other clubs were 10 %, 12 %, and 14 %, respectively, less likely to report injury than those not involved in these clubs. Research is needed to discern whether involvement is protective at the individual, interpersonal, and/or organizational level.

An additional table illustrates the progress and modification of each recommendation through the 3-Round modified-Delphi process [see Additional file 1].

## Discussion

The Delphi process (and its modifications) has demonstrated utility to reach consensus in previous research, including: program planning, needs assessment, policy determination, resource utilization, curriculum development and the development of clinical guidelines in clinical education [11, 12, 20–23]. This research project demonstrated that the Delphi process used here, modified to receive expert opinion through online survey and email [10], was a successful method for developing critical evidence-based action-oriented recommendations in the context of a national, youth injury report.

The 2 recommendations related to the relationship identified in the report between injury and food security, elicited unexpected responses from the expert panel that required further consideration by the facilitator. Expert comments indicated the importance of the research findings, the novel nature of the findings, and the relevance to policy and action, yet only half of experts agreed that these recommendations would resonate with the groups responsible to take action. Posing a different question to experts may be required to understand how these recommendations could best be utilized.

The strengths of this study include the involvement of youth as expert advisors and the strong engagement of experts as demonstrated by responses from 8 out of 9 experts to Delphi rounds 1 and 2. The decreased response rate in the final round is a known limitation for Delphi studies. However, the research team clearly invited panelists to respond to the third round only if they had further comments. It is assumed that the non-response was in fact an indication that they had no further comments or suggestions for improvement, and were satisfied with the recommendations resulting from the first 2 rounds.

Experts provided input based upon their individual expertise and understanding of the evidence. The extent to which each expert may or may not have assessed and considered prevailing political, economic, socio-cultural, environmental and other external influences is unknown, and was not formally included within this modified Delphi process.

The Delphi method has been criticized for the fact that the opinions of a small number of experts may not be representative [24]. In this study, efforts were made to select expert panelists who represented different regions, disciplines and constituent stakeholders relevant to youth injury. It is also acknowledged that the injury prevention community in Canada is relatively small and well-connected improving the chances of identifying who the generally recognized child and youth injury prevention experts are, and ensuring representativeness of the expert panel members across regions and disciplines. Experts were supported by the previous work, and preliminary list of recommendation ideas, that emanated from the 22 chapter authors of the national injury report and this expanded the diversity and number of overall contributors to the outcomes.

Poor summary and presentation of expert input by the facilitator can result in flawed synthesis between rounds [24]. In this study, the facilitator was represented by the 5 members of the research team, all of whom are expert injury prevention researchers, and familiar with the injury prevention field in Canada. This approach, that 5 minds are better than 1, is likely to have precluded flawed synthesis.

These recommendations have the potential to move the policy agenda of local governments forward with respect to child and youth injury prevention, in particular if done in partnership with the organizations and professionals listed in each recommendation. Future research to study the implementation of these recommendations and the impact on the burden of injury to children and youth in Canada would be a valuable next step.

## Conclusions

This project was successful in utilizing a modified-Delphi process to achieve agreement on 19 specific recommendations to complement the report *Injury among Young Canadians: A national study of contextual determinants.* These actions or recommendations will require multidisciplinary collaborations in order to support the proposed policy development, additional research, programming and clear decision-making for youth injury prevention.

An ideal environment is one where governments, business leaders and academics work together to ensure healthy public policy, enhance community capacity, support individual skills, and take all appropriate action to reduce the likelihood of injury and death; where society protects and nurtures high-risk members of the community and those who lack resources to fully act on their own behalf; where inequities are seen as challenges that threaten the health and safety of all, and which must be solved.

## Additional file

Additional file 1:
**Table of Recommendations changed_deleted through project.** (PDF 338 kb)

## Abbreviations

HBSC: Health Behaviour in School-aged Children Survey
CIHR: Canadian Institutes of Health Research

## References

[1] SMARTRISK (2005). Ending Canada’s Invisible Epidemic: A strategy for Injury Prevention.

[2] Discharge Abstract Database, Canadian MIS Database, Canadian Institute for Health Information, 2015.

[3] Public Health Agency of Canada. Leading causes of death, Canada, 2005, males and females combined: counts (crude death rate per 100,000). Public Health Agency of Canada. 2008. http://www.phac-aspc.gc.ca/publicat/lcd-pcd97/table1-eng.php. Accessed 6 March 2015.

[4] Freeman JG, King M, Pickett W, Craig W, Elgar F, Janssen I, Klinger D. The health of Canada’s young people: a mental health focus. Public Health Agency of Canada. 2011. http://www.phac-aspc.gc.ca/hp-ps/dca-dea/prog-ini/school-scolaire/behaviour-comportements/publications/hcyp-sjc-eng.php Accessed 6 March 2015.

[5] Currie C, Nic Gabhainn S, Godeau E (2009). International HBSC Network Coordinating Committee. The Health Behaviour in School-aged Children: WHO Collaborative Cross-National (HBSC) study: origins, concept, history and development 1982–2008. Int J Public Health.

[6] Davison CM, Russell K, Piedt S, Pike I, Pickett W and the CIHR team in Child and Youth Injury Prevention. Injury Among Young Canadians: A national study of contextual determinants. CIHR team in Child and Youth Injury Prevention. 2013. http://childinjuryprevention.ca/2013/11/1415/. Accessed 6 March 2015.

[7] Hallowell MR, Gambatese JA (2010). Qualitative Research: Application of the Delphi Method to CEM Research. J Constr Eng Manag..

[8] Linstone HA, Turoff M (eds). The Delphi Method: Techniques and Applications. New Jersey Institute of Technology. 2002. http://is.njit.edu/pubs/delphibook/index.html. Accessed 6 March 2015.

[9] FluidSurveys™. https://fluidsurveys.com/. Accessed 6 March 2015.

[10] Green A, Gance-Cleveland B, Smith A, Boebel V, Ely E, McDowell B (2014). Charting the course of pediatric nursing research: the SPN Delphi study. J Pediatr Nurs.

[11] Mertens AC, Cotter KL, Foster BM, Zebrack BJ, Hudsone MM, Eshelman D (2004). Improving health care for adult survivors of childhood cancer: recommendations from a Delphi panel of health policy experts. Health Policy.

[12] Hsu C, Sandford B. The Delphi Technique: Making Sense of Consensus. Practical Asses Res Eval. August 2007;12(10):2–8.

[13] Dalkey NC, Rourke DL, Lewis R, Snyder D (1972). Studies in the quality of life.

[14] Debecq AL, Van de Ven AH, Gustafson DH (1975). Group techniques for program planning.

[15] Ludwig B (1997). Predicting the Future: Have you considered using the Delphi Methodology?. J Ext.

[16] Okoli C, Pawlowski SD (2004). The Delphi method as a research tool: an example, design considerations and applications. Information Manag.

[17] No Regrets, Meet the Parachute Advisory Team. http://noregrets.parachutecanada.org/about-us/youth-advisory-team. Accessed 6 March 2015.

[18] Hsu C, Sandford B (2007). Minimizing Non-Response in the Delphi Process: How to Respond to Non-Response. Practical Asses Res Eval.

[19] Brooks KW (1979). Delphi technique: Expanding applications. North Central Assoc Quart.

[20] Thangaratinam S, Redman C (2005). The Delphi Technique. Obstetrician Gynaecologist.

[21] Langlands RL, Jorm AF, Kelly CM, Kitchener BA (2008). First Aid Recommendations for Psychosis: Using the Delphi Method to Gain Consensus Between Mental Health Consumers, Carers, and Clinicians. Schizophr Bull.

[22] McLeod P, Steinert Y, Meterissian S, Child S (2004). Using the Delphi process to identify the curriculum. Med Educ.

[23] Smolen JS, Aletaha D, Bijlsma JWJ, Breedveld FC, Boumpas D, Burmester G (2010). Treating rheumatoid arthritis to target: recommendations of an international task force. Ann Rheum Dis.

[24] Yousuf MI (2007). Using Expert’s Opinions Through Delphi Technique. Practical Asses Res Eval.

