# Supplementary material for: Youth injury prevention in Canada: use of the Delphi method to develop recommendations
Source: BMC Public Health. 2015 Dec 22;15:1274. doi: 10.1186/s12889-015-2600-x (PMC4687288; doi:10.1186/s12889-015-2600-x)
Supplement: Additional file 1: — Table of Recommendations changed_deleted through project. (PDF 338 kb) [file 12889_2015_2600_MOESM1_ESM.pdf]

Report Recommendations modified/deleted over the course of the development process

Note: Shading indicates that the recommendation was kept for subsequent round of the Delphi process

Suggested Recommendations (sent to panelists for 1st round of Delphi)	2nd Round of Delphi	3rd round of Delphi	Final list of 19 recommendations
1. All levels of government and sponsoring organizations are encouraged to continue to support organizations that lead injury awareness campaigns so that Canadians continue to recognize that injury remains a leading public health priority. This includes efforts by organizations to make Canadians aware of the prevalence of injury events, the nature and severity of injury among young people across the country, the numbers of young people who access the health care system because of injury, the time lost from school or other usual activities, and the economic burden.			**Recommendation 1: That federal, provincial, territorial and municipal governments, health and funding agencies make meaningful investments to support organizations that lead injury prevention initiatives in Canada.**<br>Organizations that lead injury prevention efforts must be supported. In order to prevent injuries and enhance the lives of youth, it is necessary to change public attitudes around the acceptable frequency of injury events; the risk factors and causes of injury; the types and severities of injuries; the life-long impact that injuries can have; the time lost from education and other healthy activities; the pressure that injuries add to the health care system; the economic burden to society and families; and the fact that the vast majority of injuries are preventable.
2. Federal and provincial Health Ministries as well as regional and community-level health organizations are urged to make investments to establish and continue surveillance efforts to identify new and emerging patterns of injury.			**Recommendation 2: That federal, provincial, territorial and municipal governments, health and funding agencies make meaningful investments to establish and continue comprehensive injury surveillance, inform injury prevention initiatives, monitor and evaluate outcomes and identify new and emerging patterns of youth injury.**<br>Like chronic and infectious disease surveillance, it is critical that injury trends and patterns, which include social and contextual determinants, are monitored and evaluated over time. Efforts to collaborate and share injury data and information will increase understanding and the ability to take strategic actions to reduce and prevent injuries.
3. All levels of government are strongly encouraged to support policies, programs and services that increase food security and decrease family dysfunction (and possibly poverty).<br>NB. This recommendation is based on the finding that 25% of students sampled went to school or bed hungry some of the			**Recommendation 6: That federal, provincial, territorial and municipal governments, health and funding agencies support policies, programs and services that increase food security and reduce family dysfunction.**<br>This report has noted the relationship between injuries and youth of low socio-economic status or who go to school or to bed hungry.

time and that going to bed hungry affects the number of home injuries experienced by youth, especially boys in grades 9-10. Government can look to advocacy organizations such as First Call to identify appropriate programs, policies and services.			Among boys in grades 9-10, for example, those who reported often going to school or bed hungry were four times more likely to also report one or more home injuries. Efforts to reduce injury must be multifaceted and include research (see Recommendation 17 below), food security and other family support programs.
4. Governments are urged to implement and enhance the support to non government organizations, public health agencies and mental health organizations that provide programs and services to youth with substance use issues and other high risk behaviours. Substance use can lead to impairments that leave young people vulnerable to major injury. It is also a marker for other lifestyle behaviours that lead to high injury risks. A list of evidence-based interventions can be found at the Canadian Best Practices Portal by using the keywords *youth drug and alcohol*. http://66.240.150.14/intervention/search-eng.html			**Recommendation 4: That federal, provincial, territorial and municipal governments, health and funding agencies implement policies and programs to minimize use of alcohol and drugs by youth. This includes efforts to address the culture that promotes alcohol and drug use, controlled regulation of alcohol sales, education on youth substance abuse and high-risk behaviours, and establishment of programs and services to address addictions.**<br>Services, regulations and programs that address the use and abuse of alcohol, prescription and illicit drugs are critical. Substance use can lead to increased risk-taking and impairments that leave young people vulnerable to major injury. The recreational use of drugs and alcohol among passengers and drivers of off-road vehicles is a concern particularly in rural settings. Alcohol and drug use is also a marker for other lifestyle behaviours that lead to higher risk of injury.
5. Encourage governments and non government organizations that promote active healthy living among youth to include in their public messaging that the risk of being injured at home or in the yard is not increased through participation in extracurricular activities.			
6. Provincial Education Ministries are urged to mandate evidence-based injury prevention education into the school curriculum for grades 6 to 9 that targets known risk factors (e.g. bullying, free time physical activity, poor academic performance) for injury.			**Recommendation 8: That Provincial and Territorial Education Ministries further integrate injury prevention education (e.g. bullying prevention, suicide prevention, drowning, burns, motor vehicle, sports injuries) into the school curriculum for grades 6 to 10 (and ideally from Kindergarten) and institute school policies to protect students from school-related injuries.**<br>Because children and youth spend much of their time at school, examining the relationship between characteristics of the school setting and injury risk is extremely important. The school setting is an ideal place to integrate and evaluate injury prevention efforts, since children and youth can become involved in injury prevention initiatives at school, and many activities that can lead to injuries (e.g. playground use

			participation in school sports) occur in this setting.
7. Provincial and municipal policy makers are encouraged to be proactive and institute sensible regulatory and policy solutions to recurrent injury problems where evidence exists to support such an approach to injury prevention. Examples include: infrastructure and road use policies that promote safety, helmet use laws for a variety of sports and activities, enhanced impaired driving reduction initiatives, and safe operation of off-road vehicles.			**Recommendation 3: That federal, provincial, territorial and municipal governments review, establish and enforce regulatory and evidence-informed policy solutions to prevent and control injuries.**<br>The role of government in injury prevention through the development and enforcement of good policy is essential. Examples include, but are not limited to, policies that promote training and safe operation of motor vehicles and off-road vehicles, reduction of impaired driving both on roads and in off-road situations, helmet use during wheeled activities, skiing, and snowboarding, and concussion prevention and management.
8. National, provincial, regional and community-level injury prevention professional and research networks (e.g. the Canadian Collaborating Centres on Injury Prevention) should collaborate with colleagues in health promotion, physical activity, and those representing sport and recreation bodies in an effort to determine the correct balance in promoting healthy physical activity, without placing children at undue risk of unintentional injury.			**Recommendation 10: That the Canadian Collaborating Centres for Injury Prevention and Parachute Canada partner and collaborate with Active Healthy Kids Canada, ParticipACTION, neighbourhood youth serving agencies and coaches to integrate injury prevention into the promotion of healthy physical activity for children and youth.**<br>Physical activity and play are essential to healthy development among children and youth, but may be accompanied by increased risk of injury. Collaborations between those promoting physical activity and play, and injury prevention partners are essential to ensure safe, yet stimulating environments for healthy development. Examples include: redesigning school and community playing fields, play spaces and active transportation routes; informing school policies regarding bullying and supervision; providing return-to-play guidelines; providing sport-specific injury prevention; and integrating bullying interventions into physical activity promotion.
9. Communicate to parents (as they may be relieved to know) that no relationship was found between home injury and the length of time a student lived in Canada, weekend screen time, or participation in extracurricular activities.			
10. Urge Parachute Canada and injury researchers to collaborate with medical professionals and youth serving organizations in an effort to fully understand the type and causes of home injuries, as well as the underlying risk factors among grades 9 and 10 girls who use illicit and prescription drugs. And, to develop, implement and evaluate injury prevention initiatives based on this understanding.			

11. Urge Parachute Canada and injury researchers to collaborate with medical professionals and social workers to fully understand the type of injuries and underlying risk factors that boys in foster homes experience. And, to develop, implement and evaluate injury prevention initiatives based on this understanding.			**Recommendation 7: That injury researchers collaborate with medical and social welfare professionals to better understand injury risks and social disparity risk factors leading to home injuries, particularly among youth in foster care, and develop concrete recommendations for injury prevention initiatives based on this understanding.** Approximately 12% of all youth injuries occur within the home and yard setting and not all youth are at equal risk of sustaining a home-based injury (see recommendation 6). A greater proportion of younger girls and older boys residing in group homes or foster care, for example, reported more injuries and severe injuries than their peers who were not in foster care. Injury prevention efforts need to be informed by greater understanding of the factors leading to these home injuries.
12. Public health nurses, police liaison officers, Parent Advisory Councils and other community programs are encouraged to implement evidence-based injury prevention programs that target students in grades 6-8 and their families. A list of evidence-based interventions can be found at the Canadian Best Practices Portal by using keywords such as *injury prevention, bullying, alcohol, drugs*, etc. http://66.240.150.14/intervention/search-eng.html			
13. In partnership with municipalities and community and neighbourhood associations, public health and non-governmental organizations should continue to implement and tailor injury prevention efforts to target local high-risk communities.			**Recommendation 9: That public health personnel, police liaison officers, Parent Advisory Councils, municipalities and other community and neighbourhood agencies review and understand the scope of youth injury in their community in order to implement and evaluate evidence-based injury prevention policies, programs and initiatives.** Every community has groups who are at higher risk for injury, such as youth from lower socio-economic status families or boys from affluent families who engage in high-risk sporting activities. The local injury stakeholders listed above need to work together with the researcher community to target and evaluate injury prevention initiatives towards high-risk groups.
14. Municipalities, neighbourhoods, community groups, Parks and Recreation and schools are urged to create safe physical environments where youth can spend time. This ranges from increasing the number of parks youth can access, to upgrading vacant or shabby buildings. This can positively influence sense			**Recommendation 11: That municipalities, community and neighbourhood leaders, Parks and Recreation and schools work with youth to create safe physical environments where youth want to spend time.** Development of spaces for physical activity and play can positively

of community and foster trust in neighbourhood, which is also important for injury reduction. In addition, the provision of safe environments promoting physical activity can have positive influences on other health outcomes such as obesity.			influence sense of community and foster trust in neighbourhood, which is important for injury reduction. In addition, the provision of environments promoting physical activity can have positive influences on other health outcomes such as obesity.
15. Municipalities, schools and agencies that design and create environments for youth must ensure that steps are taken to develop spaces that include age-appropriate challenge and risk, while at the same time removing injury hazards. If we are to fully understand the determinants of health and ill-health, it is important to take an ecological approach and look to the environments or "contexts" where youth spend their time.			
16. Youth, parents and youth serving organizations as well as municipalities, neighbourhoods, community groups, Parks and Recreation and schools are encouraged to engage with multiple stakeholders and partners to create change that fosters community cohesion through community events, clubs, or activities. This should be measured through perceptions of neighbourhood safety.			
17. Youth, parents and youth serving organizations as well as municipalities, neighbourhoods, community groups, Parks and Recreation and schools should target other high-risk groups with evidence-based injury prevention efforts such as boys from the most affluent families who have high rates of severe injuries, probably due to their participation rate to high-risk sports activities.			
18. Public health nurses, police liaison officers, Parent Advisory Councils, other community level parent programs, school districts, schools, youth serving organizations as well as parents and youth themselves must continue to support and advocate for drug and alcohol prevention programs and harm reduction programs as a protection against injury. Evidence-based programming that is collaborative with youth is recommended. A list of evidence-based interventions can be found at the Canadian Best Practices Portal by using keywords *youth drug and alcohol*. http://66.240.150.14/intervention/search-eng.html			**Recommendation 5: That public health personnel, police liaison officers, Parent Advisory Councils, school districts, municipalities and other community and neighbourhood youth serving agencies continue to support and advocate for programs and policies aimed at preventing youth drug and alcohol abuse and promoting associated harm reduction programs as a protection against injury.** Injuries were more common among youth who reported illicit or prescription drug use, binge drinking or whose friends abused drugs or alcohol. Public health personnel, police liaison officers, Parent Advisory Councils, school districts, municipalities and youth servicing agencies can assist governments and funding agencies by advocating for, leading or supporting initiatives to address substance use.

19. Schools and school districts are urged to implement and enhance evidence-based anti-bullying and anti-violence programs that target the perpetrator, victim and bystanders, and which include school administrators in the delivery of appropriate consequences for bullying behaviours. A list of evidence-based interventions can be found at the Canadian Best Practices Portal by using the keywords *bullying* or *fighting*. http://66.240.150.14/intervention/search-eng.html			
20. School districts, schools and Parent Advisory Councils are encouraged to celebrate and continue to advocate for ongoing anti-bullying programs so as to continue to reduce the risk of injury within the school setting.			**Recommendation 12: That school districts, school administrators and Parent Advisory Councils review, continue to implement and support anti-bullying and anti-violence policies and programs that target perpetrators, victims and bystanders.** This report found that children and youth who reported being bullied were up to twice as likely to be injured than those who did not report being bullied. This association was particularly strong for boys in grades 6-8 and girls in grades 9-10. School administrators must lead in the delivery of appropriate consequences for bullying behaviours.
21. School districts, schools and Parent Advisory Councils are encouraged to implement evidence-based peer-mentorship programs that address the social context of the school environment and improve feelings of belonging and safety. A list of evidence-based interventions can be found at the Canadian Best Practices Portal by using keywords such as *youth relationships, bullying*, etc. http://66.240.150.14/intervention/search-eng.html			**Recommendation 13: That school districts, school administrators, Parent Advisory Councils and youth leadership groups implement peer-mentorship programs that address the social context of the school environment and improve feelings of belonging and safety.** This report found a relationship between injury and emotional well-being, such as not feeling respected or not belonging at school. Schools must work together with local youth serving agencies, Parent Advisory Councils and youth to create a culture that fosters inclusiveness, respect and improves tolerance of differences/diversity and discourages bullying.
22. Research funding bodies need to support further research so as to understand the fundamental determinants of injury, including individual risk behaviours, as well as context and environments.			**Recommendation 14: That research funding bodies support programs of research that seek to understand the fundamental determinants of youth injury, including surveillance to identify new patterns and trends, and interventions that target specific high risk and/or vulnerable populations, contextual determinants and risk and protective factors.** While this report provides some insights into the relationship between social and contextual determinants, risk and protective factors, and youth injury, more research is needed to enhance our understanding.

			Recommendations 15 to 19 are directed at the injury research community, including academic and applied researchers, policy makers and practitioners to develop innovative research projects that shed light on the following specific areas in relation to injury: substance use, high-risk youth, going to bed hungry, peer relationships and involvement in a sport club, youth club or voluntary service.
23. Research funding bodies need to support further research on the recreational use of prescription drugs, and its impact on injury patterns and rates. This is an increasingly important risk factor for adolescent injury.			**Recommendation 15: That research programs are developed and supported to improve understanding of the culture that promotes the use of alcohol, illicit and prescription drugs for recreational purposes, and the impact on child and youth injury patterns and rates.**<br>As stated in recommendations 4 and 5, alcohol, illicit drug and prescription drug use are increasingly important risk factors for adolescent injury. Further research will aid our understanding of where and how to intervene.
24. In order to develop optimal methods to prevent injury among vulnerable, high-risk youth, including those from rural and remote regions, research funding bodies need to make additional investments in studies that illuminate recurrent determinants and patterns of injury in these populations.			**Recommendation 16: That research programs are developed and supported to implement optimal methods to prevent injury among high-risk and/or vulnerable youth, including those from rural and remote regions; investigate recurrent determinants and patterns of injury; social disparities; and, risk and protective factors.**<br>The risk of injury is not the same for all children and youth as some characteristics and behaviours increase or decrease risks for injury. In addition to level of family function, being bullied, involvement in high-risk sports, and use of alcohol, illicit and prescription drugs, this report found that the proportion of students reporting an injury increased as the population size of the city or town where they attended school decreased. Further research and evaluation is needed to inform targeted interventions for all high-risk children and youth, including those living in rural locations (<1,000 population).
25. Research funding bodies need to support studies that shed light on the relationships between going to school or bed hungry and injury so as to fully understand why going to school or bed hungry increases youth risk of being injured.			**Recommendation 17: That research programs are developed and supported to investigate the relationships between going to school or bed hungry and the increased risk for injury among youth, with a particular focus on policy solutions.**<br>Understanding of the relationships between food insecurity, family dysfunction and injury among youth will allow solutions to focus on some of these root causes.
26. Research funding bodies are strongly urged to support research that improves understanding of peer relationships			**Recommendation 18: That research programs are developed and supported to illuminate understanding of the effects of various child**

among youth. Specifcally,<br>a) Explore why having close male friends was a risk factor for girls and younger boys, but protective for older boys<br>b) Explore attitudes and beliefs towards friends, relational aspects within friendships, as well as the discourse between friends and how this relates to risk and injury<br>c) Study the changes that may occur within friendships during the transition from middle to high school, when the influence appears to shift to become a protective factor for injury among boys<br>d) Investigate the relative impact of the number of friends compared to relational aspects of friendships, on injury risk<br>e) Investigate the processes within friendships that increase injury risk. It is evident that peers do engage in risk behaviours together, but a more systematic investigation of the relationship between peer contact and risk taking could be informative.			**and youth peer relationships and peer activities on injury risk.**<br>The current findings suggest that there is merit in exploring social and contextual factors when creating injury prevention programming. Among girls, having close male friends increases the risk for injury; however, having close male friends is a protective against the occurrence of injury among older boys. As risk factors for injury vary for boys and girls, further research and evaluation is needed to inform targeted interventions.
27. Research funding bodies need to support research to explore understanding of youth involvement in sports clubs or other clubs as a protective factor against injury. This research needs to discern whether involvement is protective because of something attributable to the youth themselves (e.g., temperament, personality, socio-economic factors) or whether it reduces exposure to higher risk contexts (i.e., less structured and unsupervised) or provides opportunity for relationships with youth having attributes that reduce injury risk (e.g., personality characteristics such as inhibitory control).			**Recommendation 19: That research programs are developed and supported to understand child and youth involvement in sports and social clubs as a protective factor against injury.**<br>Children and youth involved in voluntary service, youth clubs, and other clubs were 10%, 12%, and 14%, respectively, less likely to report injury than those not involved in these clubs. Research is needed to discern whether involvement is protective at the individual, interpersonal, and/or organizational level.
